# 

**Published:** 1896-12

**Authors:** 


### CONTENTS.

ORIGINAL ARTICLES:
Inaugural Address, New York State Veterinary College. By PROF. JAMES LAW . 805
Parturient Apoplexy By J. CURTIS MICHENER . . . . . . . . . 813
Our Profession. By F. W. HOPKINS, D.V.S. . . . . . . . . 820
A Peculiar Case of Hybridism. Reported by DR. CECIL FRENCH . . . 822
ABSTRACTS FROM FOREIGN JOURNALS:
Typhoid Infection in a Two and a Half Year Old Child from Drinking Milk not thoroughly Boiled—Petroleum Benzine as an Anæsthetic—To Remove Rust from Instruments—Deaths Caused by Malignant Pustule and Lyssa in Italy in 1893 and 1894—The Disinfection of Infected Wounds by Means of Septic Materials—Some Remarks on the Effects of Drugs—Treatment of Cancer with Cancer-serum (Erysipelas-serum) . . . . . . . . . 823–826
EDITORIALS:
Welcome, 1897—To Our Readers—Senate Bill No. 1552—Army Legislation—The Scent of Spoils—Our Buffalo Resolutions on Tuberculosis . . . 831–835
NECROLOGY—MARRIAGES . . . . . . . . . . . . . . 836
CONTROL WORK:
Australia—Jamaica—Indiana—Pennsylvania—New York—Vermont—Massachusetts—Illinois . . . . . . . . . . . . . 837–838
CORRESPONDENCE—EDUCATIONAL—USEFUL FORMULAS . . 839–841
SOCIETY PROCEEDINGS:
Keystone Veterinary Medical Association—Chicago Veterinary Society—Niagara and Orleans County Veterinary Medical Society—Montreal Veterinary Medical Association—Veterinary Medical Society of New York County—Colorado State Veterinary Medical Association—Pennsylvania State Board of Veterinary Medical Examiners . . . . . . . . . . . . . . . 843–851

